# Testing the differential effects of acceptance and attention-based psychological interventions on intrusive thoughts and worry

**DOI:** 10.1016/j.brat.2017.01.012

**Published:** 2017-04

**Authors:** B. Ainsworth, H. Bolderston, M. Garner

**Affiliations:** a Psychology. Faculty of Social, Human & Mathematical Sciences, University of Southampton, UK; b Primary Care and Population Sciences, Faculty of Medicine, University of Southampton, UK; c Research Centre for Behaviour Change, Department of Psychology, Bournemouth University, UK; d Clinical and Experimental Sciences, Faculty of Medicine, University of Southampton, UK

**Keywords:** Anxiety, Attention, Acceptance, Mindfulness, Meditation, Worry, MBCT, mindfulness-based cognitive therapy, PMR, progressive muscle relaxation, FA, focused attention, OM, open-monitoring meditation, VAS, visual analogue scales

## Abstract

**Background:**

Worry is a key component of anxiety and may be an effective target for therapeutic intervention. We compared two psychological processes (attention and acceptance) on the frequency of intrusive worrying thoughts in an experimental worry task.

**Method:**

77 participants were randomised across three groups and completed either a 10 min attention or acceptance-based psychological exercise, or progressive muscle relaxation control. We subsequently measured anxiety, and the content and frequency of intrusive thoughts before and after a ‘worry induction task’.

**Results:**

Groups did not differ in baseline worry, anxiety or thought intrusions. Both attention and acceptance-based groups experienced fewer negative thought intrusions (post-worry) compared to the relaxation control group. The acceptance exercise had the largest effect, preventing ‘worry induction’. Increases in negative intrusive thoughts predicted subjective anxiety.

**Discussion:**

We provide evidence that acceptance and attention psychological exercises may reduce anxiety by reducing the negative thought intrusions that characterise worry.

## Highlights

• We used an experimental worry task to see how psychological interventions can target worry.
• We compared 10-min attention and acceptance-based exercises to progressive muscle relaxation.
• Both acceptance and attention were better than relaxation, and acceptance had the largest effect.
• Increases in negative intrusive thoughts predicted subjective anxiety across all groups.
• Acceptance and attention psychological exercises may help anxiety by reducing worry.

## Graphical abstract

**Figure:**
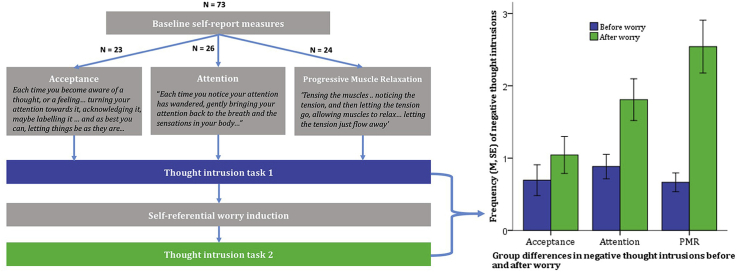

## 1 Introduction

Maladaptive cognitive and attentional biases are implicated in the etiology and maintenance of anxiety. Anxious individuals preferentially attend to threat (e.g. Mogg, Garner, & Bradley, 2007) and interpret ambiguous information in a threatening manner (e.g. Castillo & Leandro, 2010; see Bar-Haim, Lamy, Pergamin, Bakermans-Kranenburg, & van Ijzendoorn, 2007; for a review). Individuals with anxiety also have broader deficits in attention control and executive processing, are more readily distracted and unable to focus on top-down ‘goal-directed’ tasks, instead devoting limited attentional resources to negative distractors (e.g. Attention Control Theory, Eysenck et al., 2007, see recent integrative review by Mogg & Bradley, 2016).

The cardinal symptom in generalized anxiety—worry—is characterised by increased frequency and uncontrollability of distracting negative thoughts about current and future threat (see Hirsch et al., 2015, Raedt et al., 2015). These recurrent thoughts comprise verbal problem-solving of perceived future threat, and may be accompanied with distressing imagery. Active worry limits processing resources required to complete tasks efficiently (Ouimet, Gawronski, & Dozois, 2009) and can persist in anxious individuals due to anxiety-related deficits in attention control, and reinforcing beliefs that worry is adaptive (Borkovec, Robinson, Pruzinsky, & DePree, 1983). Consequently, mechanisms that exacerbate and maintain worry can be considered as putative targets for therapeutic intervention in anxiety (Gaynor, 2014, Hirsch et al., 2015, Wiers et al., 2013).

The relationship between poor attention control and negative intrusive thoughts (i.e. worry) in anxious individuals has been established - increased self-reported worry is associated with poorer performance on an attentional flanker task, likewise improved attention control (flanker performance) is associated with fewer worry-related intrusive thoughts (Fox, Dutton, Yates, Georgiou, & Mouchlianitis, 2015). Furthermore, experimental methods that directly reduce attentional bias to negative information (attention bias modification) can reduce negative thought intrusions following a period of active worry (Hirsch et al., 2011).

Mindfulness meditation encourages deliberate, nonjudgemental attention to internal and external stimuli in the present moment (Williams & Kabat-Zinn, 2011), and offers promise as a cost-effective treatment for anxiety (Hofmann, Swyer, Witt & Oh, 2010). Mindfulness exercises typically target two processes – attention and acceptance (Bishop et al., 2006). Attention involves paying ‘objective’ attention to internal and external stimuli while acceptance encourages having open and receptive attention to on-going experiences (Brown & Ryan, 2004). Recent neuropsychological perspectives of mindfulness outline a range of ‘bottom-up’ and volitional ‘top-down’ mechanisms of action that might usefully target negative thought intrusions, persistent worry and anxiety (see Holzel et al., 2011). Despite this there have been few attempts to operationalize, manipulate and compare acceptance and attention component processes, to dissociate their therapeutic effects and optimise allied treatment protocols.

Recent laboratory studies suggest that mindfulness exercises that target attention and open-monitoring can improve attention control (Ainsworth et al., 2013, Zeidan et al., 2010, Jha et al., 2007), emotion regulation (Burton, Schmertz, Price, Masuda & Anderson, 2013) and reduce experimentally induced anxiety (e.g. subjective anxiety during carbon-dioxide challenge, Ainsworth et al., 2015). Mindfulness meditation has been shown to reduce self-reported worry in non-clinical high worriers (Delgado-Pastor et al., 2015), in patients undergoing a mindfulness-based cognitive therapy (MBCT) for relapse prevention of recurrent depression (Ietsugu et al., 2015), and in individuals with generalized anxiety disorder (GAD) taking part in an internet delivered acceptance-based therapy (Dahlin, Ryberg, Vernmark & Annas, 2016). Similarly, there is evidence that mindfulness-based interventions can increase measures of acceptance (Schroevers & Brandsma, 2010).

However, the growing popularity of mindfulness meditation raises the question of whether mindfulness might usefully target unwanted thought intrusions and worries in the wider non-clinical population. The existing literature further suggests that acceptance and attention training may have differential effects on such processes. Evidence that experiential avoidance (unwillingness to experience negative thoughts and emotions) is associated with problematic worry (Buhr and Dugas, 2009, Roemer et al., 2005) supports positive effects of interventions specifically designed to increase acceptance of anxiety and worry (e.g. Roemer & Orsillo, 2007). This contrasts with the mixed therapeutic effects of interventions that target discrete attentional biases e.g. threat avoidance attention training (see review in Mogg & Bradley, 2016).

We compared two psychological processes that feature in contemporary mindfulness-based interventions, attention and acceptance, on the frequency of intrusive worrying thoughts in an experimental worry task. We measured the frequency and valence of thought intrusions using an established measure of thought intrusions developed by Ruscio and Borkovec (2004) and adapted by Hirsch, Hayes, and Mathews (2009). This task has been widely used to examine thought intrusions and worry in non-clinical groups (Krebs, Hirsch, & Mathews, 2010), individuals with elevated worry, and generalized anxiety disorder (Hayes et al., 2010, Hirsch et al., 2009) and associated conditions (i.e. insomnia symptoms, Baker, Baldwin, & Garner, 2015). The thought intrusions task measures the occurrence of resting level, spontaneously occurring thoughts which distract from the current task. It further measures the pervasiveness of thought intrusions following a period of active worry on a topic chosen by the participant. This paradigm overcomes limitations associated with self-report questionnaires that ask participants to retrospectively report the frequency with which they worry about a pre-determined set of topics chosen by researchers, and that might be confounded by recall bias.

Our study compared the effects of attention and acceptance-based practices vs. progressive muscle relaxation (PMR)—an active control condition—on negative thought intrusions before and after a worry-induction (see Hirsch et al., 2009). We predicted that acceptance and attention exercises (compared to PMR control) would reduce negative thought intrusions and subjective anxiety after worry induction. Further, we predicted that the acceptance exercise would be superior to the attention exercise by encouraging broader acceptance of private thought/emotions and reducing experiential avoidance that may otherwise sustain worry. Finally, following cognitive models of worry we examined whether reduced negative thought intrusions would be associated with reduced anxiety.

## 2 Method

### 2.1 Participants

An unselected sample of 77 volunteers (56 female/21 male; *M*_age_ = 20.8, *SD*_age_ = 3.2) were recruited from local adverts on a university campus and surrounding area, and randomly allocated to one of two mindfulness groups (focused attention: FA, or open-monitoring acceptance, OM) or an active control (progressive muscle relaxation: PMR). Sample size was calculated a priori to detect effects similar to those observed in previous studies that have used this self-referential worry task (e.g. Hayes et al., 2010: between-group difference on negative thought intrusions at post-test, *f*^*2*^ = 0.43). Informed consent was received from all participants before taking part in the study. Participants received course-credits or £6 money in return for participation. Participants were from a range of ethnicities: 70% British, 11% other white, 5% Chinese, 4% other Asian, 4% Indian, 3% African, 3% other. Participants rated their prior experience of mindfulness, on a scale ranging from 1 (I have never heard of mindfulness) to 6 (I regularly practice mindfulness). Current practitioners (score = 6) were removed (n = 4). Final analysis consisted of 73 participants: acceptance-based (N = 23, *M*_age_ = 21.1, *SD*_*age*_ = 3.6, 16 female), attention-based (N = 26, *M*_age_ = 20.6, *SD*_*age*_ = 2.3, 20 female), PMR (N = 24, *M*_age_ = 20.3, *SD*_*age*_ = 3.5, 18 female). Groups did not differ on age, gender, nor measures of dispositional mood, baseline state mood or mindfulness, see Table 1.

### 2.2 Measures

#### 2.2.1 Self-report measures

Participants completed established self-report measures of trait anxiety (Spielberger State-Trait Anxiety Inventory; Spielberger, Gorusch, Lushene, Vagg, & Jacobs, 1983), attention control (Attention Control Scale, comprising measures of dispositional/trait abilities to focus attention, limit distraction and volitionally/flexibly shift attention; α = 0.75; Derryberry & Reed, 2002), mindfulness (Philadelphia Mindfulness Scale; comprising items that measure dispotional/trait present-centred awareness/attention and acceptance; α = 0.72; Cardaciotto, Herbert, Forman, Moitra, & Farrow, 2008) and worry (Penn-State Worry Questionnaire; Meyer, Miller, Metzger, & Borkovec, 1990).

#### 2.2.2 Self-report anxiety ratings

Visual analogue ratings (VAS scales) quantified the extent that participants experienced anxiety (‘anxious’, ‘nervous’, ‘worried’) by asking them to rate their current state on a Likert-scale from 0 (Not at all) to 100 (Extremely). Measures of current anxiety were taken before the intervention (baseline), after the intervention/before the worry task (post-int), and after the worry task (post-worry).

#### 2.2.3 Thought intrusions task

Consistent with Hirsch et al. (2009) the thought intrusions task contained three stages: an initial 5 min breathing focus, a 5 min worry period and a 5 min post-worry breathing focus. During pre- and post-worry periods, participants were instructed to focus their attention on their breathing throughout. After an initial practice period (lasting 30 s) participants were told that they would hear 12 computer-generated tones during a 5-min period. On hearing each tone, participants were asked to either state whether they were focusing on their breathing, or describe any intrusive thought and whether it was positive, negative or neutral (e.g. “Looking forward to seeing my friends; positive”, “Worried I won't meet my deadline; negative”). The experimenter logged all thought intrusions as they occurred and confirmed the reported valence with the participant at the end of the study (generating scores of total thought intrusions [0–12] as well as totals for the number of negative, neutral and positive intrusions). Between pre- and post-worry breathing focus periods, participants were asked to identify a current worry topic. They briefly outlined the topic to the experimenter (to confirm it was not depressogenic and referred to a negative future occurrence) and asked to think about the worry topic for 5 min while the experimenter left the room. After 5 min the experimenter returned to the room and participants rated the worry from 0 (Not at all) to 100 (Extremely) on how catastrophic it would be, how likely it was to occur and how well they would cope with it, before completing the post-worry breathing focus task. One-way ANOVA suggest that groups did not differ in their subjective assessment of their worry-topic (see Table 1).

### 2.3 Guided acceptance and attention-based interventions

Each 10-min intervention was developed by an experienced clinical psychologist (HB) with expertise in delivering mindfulness-based interventions (since 1998). Participants were instructed to listen to a 10-min guided-meditation audio-recording. The experimenter left the room while participants completed the practice.

#### 2.3.1 Acceptance

During open-monitoring and acceptance meditation, participants were guided to accept thoughts, feelings and other kinds of private experiences, such as physical sensations. *“Direct your attention inwardly… notice thoughts, emotions, physical sensations… any other kinds of experiences as they show up in the field of your awareness… sitting and noticing what's here, right now, for you…. Each time you become aware of a private experience, such as a thought, or a feeling… turning your attention towards it, acknowledging it, maybe labelling it … and as best you can, letting things be as they are … making space for your experiences.”*

#### 2.3.2 Attention

In focused attention (FA) meditation, participants were asked to focus their attention solely towards a specific physical sensation, and regain this focus whenever it was lost. Participants were instructed to *“Become aware of the sensation of breathing… noticing where in the body the physical sensations of breathing are vivid for you, right now… choosing one place to follow the breath… making a decision to stay with this place… bringing your attention and your curiosity to each breath… Feeling the moment-by-moment physical sensations as you breathe in and breathe out. And each time you notice your attention has wandered, gently bringing your attention back to the breath and the sensations in your body…”*

#### 2.3.3 Progressive muscle relaxation

PMR was used as an active control intervention. PMR has been found to be an effective method of stress-reduction in single session and short-term interventions (Agee et al., 2009, Rausch et al., 2006), and has previously used as an active control against which to evaluate mindfulness and component processes (Jain et al., 2007). During PMR practice, participants were asked to ‘*develop the skills of relaxing groups of muscles where you may be carrying tension’*, first by *‘taking a couple of slow, deep breaths, in and out’* and then by tensing the muscles in their toes, feet and lower legs, before being asked to *‘feel the tension in your toes, feet and lower legs'… ‘hold the tension, and then breath out, and let the muscles relax’ … ’notice the difference between when they were tense, and now they are relaxed’*. Participants continued to practise this method throughout the body . The PMR intervention was developed to mirror the volume, pace and audio dynamic of the attention and acceptance interventions, with equivalent periods of guidance and silence.

## 3 Results

### 3.1 Effects of acceptance, attention and relaxation on experimentally induced worry

A mixed-model ANCOVA examined the effects of Group (Acceptance vs. Attention vs. PMR) x Emotion (negative vs. neutral vs. positive) x Time (pre vs. post-worry) on the frequency of thought intrusions, with age, gender, worry topic ratings and previous mindfulness exposure included as covariates.^1^ A three-way interaction [*F*_(4,128)_ = 3.50, *p* = 0.01, *η*_*p*_^2^ = 0.10] was explored using a separate ANCOVA for each emotion (Table 2).

**Fig. 1:**
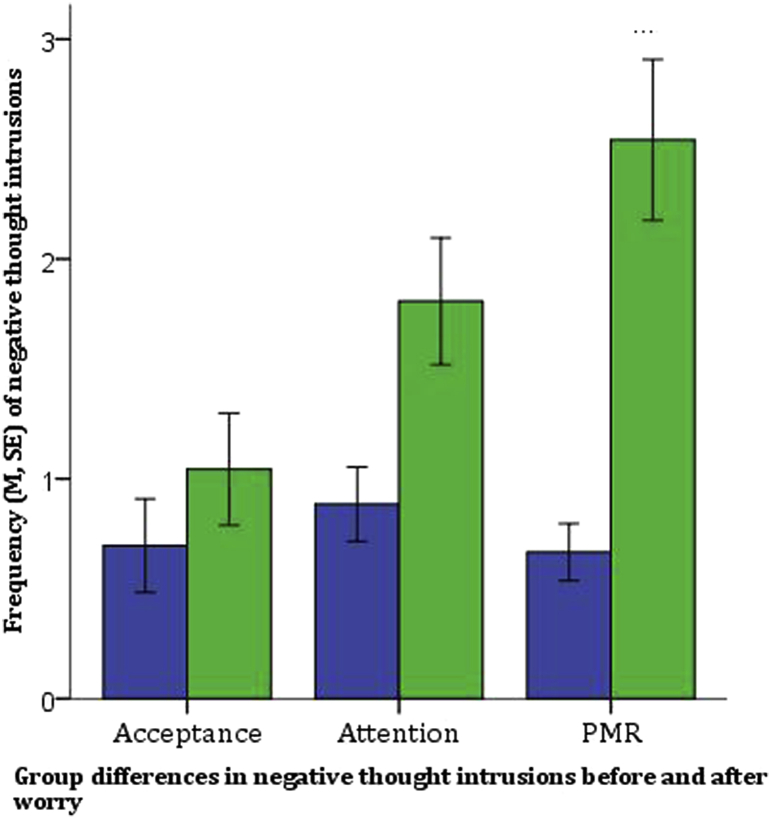
Group differences in negative thought intrusions before and after worry.

An interaction between time and group on the frequency of negative thought intrusions *[F*_(2,64)_ = 9.23, *p* < 0.001, *η*_*p*_^*2*^ = 0.22, see Fig. 1] was characterised by less induced worry in the acceptance compared to both the attention group [*F*_(1,41)_ = 4.16, *p* = 0.048, *η*_*p*_^*2*^ = 0.09] and PMR group [*F*_(1,39)_ = 15.70, *p* < 0.001, *η*_*p*_^*2*^ = 0.29], and less induced worry in the attention group than PMR group. [*F*_(1,42)_ = 6.46, *p* = 0.01, *η*_*p*_^*2*^ = 0.13].

Paired t-tests examined changes in negative thought intrusions within each group. While frequency of negative intrusions did not increase in the acceptance group [*M*_*diff*_ = 0.35, *t*_*(22)*_ = 1.70, *p* = 0.10], there was a moderate increase in the focused attention group [*M*_*diff*_ = 0.92, *t*_*(25)*_ = 3.27, *p* = 0.003, *g*_*av*_ = 0.75] and a large increase in the progressive muscle relaxation control group [*M*_*diff*_ = 1.88, *t*_*(23)*_ = 5.96, *p* < 0.001, *g*_*av*_ = 1.38].

There were no main nor interaction effects on the frequency of positive intrusions [*F*s < 0.39, *p*s > 0.68], nor on the frequency of neutral intrusions, [*F*s < 1.68, *p*s > 0.20].

### 3.2 Effect of worry induction on subjective anxiety

Participants' subjective anxiety scores (averaged across VAS items: ‘anxious’, ‘nervous’ and ‘worry’) were entered into a 3 (group: acceptance vs. attention vs. PMR) x 3 (time: baseline vs. post-intervention vs. post-worry) ANCOVA. A main effect of time [*F*_(2,128)_ = 6.98, *p* = 0.001, *η*_*p*_^*2*^ = 0.10] was characterised by increased anxiety following worry induction [*M* = 55.1, *SD* = 19.5] compared to baseline [*M* = 26.8, *SD* = 19.5] and post-intervention [*M* = 17.3, *SD* = 16.7], irrespective of group [*F*_(4,128)_ = 1.60, *p* = 0.18].

### 3.3 Associations between worry-induced thought intrusions and subjective anxiety

Linear regression found that anxiety after worry induction (controlling for pre-induction anxiety, age, gender, mindfulness experience and worry content) was predicted by the increase in negative thought intrusions (*Beta* = 0.29, *t* = 2.80, p = 0.007).

**Fig. 2:**
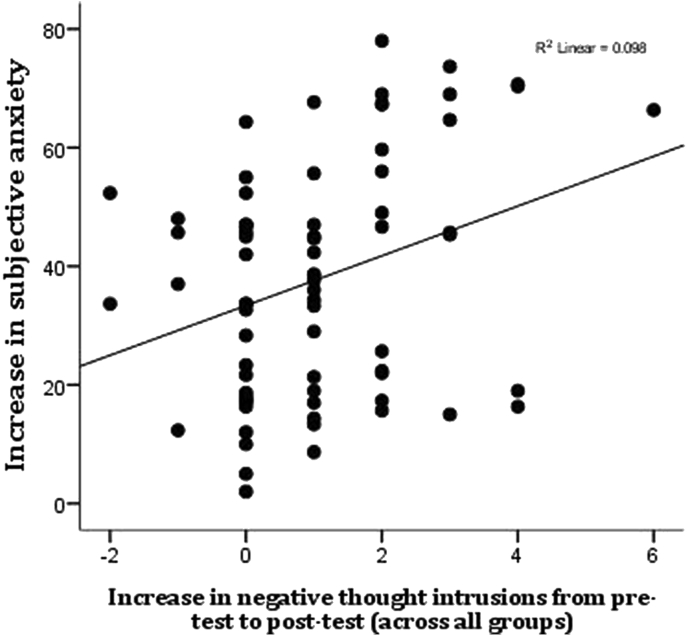
Associations between increases in negative thought intrusions and self-report anxiety.

Increases in anxiety were associated with greater negative thought intrusions (*r* = 0.31, *p* = 0.007; see Fig. 2), but not positive or neutral intrusions [*r*s > −0.07, *p*s > 0.54].

## 4 Discussion

In our study, acceptance-based mindfulness is more effective than attention-based mindfulness at restricting negative thought-intrusions following a period of active worry compared to relaxation control. Both acceptance and attention-based interventions were superior to progressive muscle relaxation.

Groups did not differ in baseline worry, anxiety or thought intrusions, nor baseline thought intrusions (post intervention but pre-worry). Instead, results suggest protective effects of acceptance and to a lesser extent attention-based exercises, that reduce the persistence of negative worrisome thoughts (beyond the worry challenge). Subgroup analyses provide evidence that these interventions reduce worry in both high and low worriers. Consequently these findings suggest acceptance (and attention) might be well placed to benefit individuals who may be vulnerable to repetitive maladaptive thoughts about anticipated negative life events (i.e. those reporting high levels of trait worry), and are in line with previous research demonstrating a strong anxiolytic effect of open-monitoring meditation compared to a more modest effect of focused attention in experimental models of anxiety (Ainsworth et al., 2015). However in the current study we sought to target acceptance alongside open-monitoring to conceptually and operationally better differentiate the acute effects of acceptance and focused attention component processes. Both interventions were well tolerated by an unselected sample of participants drawn from a young adult population, and suggest similar exercises might be adopted in remote/on-line interventions to help reduce persistent worry.

Evidence that acceptance can mitigate negative thought intrusions to a greater extent than attention-based practice supports the value of treatment approaches that emphasise acceptance of private experiences, such as Acceptance and Commitment Therapy (Hayes, Strosahl, & Wilson, 2012). Our findings also highlight acceptance as an important mechanism (and to a lesser extent attention) through which existing mindfulness interventions might reduce persistent patterns of negative thinking that increase risk of relapse of mood and anxiety disorder e.g. rumination in depression, and prospective worry in generalized anxiety disorder (e.g. Ietsugu et al., 2015). Our study was not designed, nor powered, to examine associations between self-report anxiety, worry, and thought intrusions frequency/content within each group. Nevertheless we found some evidence (across the sample) that increases in negative thought intrusions were associated with subsequent increases in subjective anxiety, consistent with cognitive models of pathological worry in anxiety (Hirsch & Mathews, 2012). This finding fits with dual-process models of anxiety (Ouimet et al., 2009) that suggest general deficits in attention control and hypersensitivity to threat interact to increase distraction, prioritise negative cognitions/stimuli, impair goal-directed processing and increase/persist anxiety.

Our findings support the use of acceptance and attention based interventions to increase resilience against repetitive negative thoughts and associated anxiety. To this end, acceptance may be particularly well placed to help individuals regulate typical everyday worries before they become catastrophic, repetitive and debilitating. Furthermore acceptance-based interventions may augment attention interventions that focus on discrete attention mechanisms to improve therapeutic outcomes (Mogg & Bradley, 2016). However, we note that our interventions did not reduce subjective anxiety (despite effects of acceptance and attention on negative thought intrusions, and associations between increased negative thought intrusions and anxiety). Consequently future studies should test whether extended practice (expertise) translates positive reductions in worrisome thoughts to therapeutic reductions in subjective anxiety. Furthermore, we might adapt experimental sampling measures of worry/thought intrusions (used here) to examine the extent to which acute reductions in worry (shown here) persist over time and context e.g. using mobile phones/apps to sample mood and cognition (Killingsworth & Gilbert, 2010). Consequently, future studies could extend our preliminary positive but acute effects in unselected young adults to populations at risk of persistent worry e.g. primary and secondary healthcare and those with chronic physical health conditions (Thomas, Bruton, Moffatt, & Cleland, 2011). Future longitudinal studies would then be better able to take baseline pre-intervention measures of ‘thought intrusions’ to quantify within-subject change (in addition to group differences in post-intervention worry change scores observed in our study). These studies will continue to require valid active control groups against which to evaluate proposed therapeutic mechanisms of action, control non-specific effects and improve participant/experimenter blinding (see Ainsworth et al., 2013; for more information see MacCoon et al., 2012).

Our findings provide evidence that acceptance and attention-based interventions can reduce worry about future negative life events in non-clinical individuals. These psychological exercises may in the longer-term moderate anxiety by reducing the persistent negative thought intrusions that characterise worry. Consequently future studies should examine their utility as part of accessible, cost effective, low intensity interventions for individuals experiencing repetitive, unwanted intrusive thoughts.

## Author contribution

All authors developed the study concept and design. HB developed the interventions. BA completed testing and data collection and data analysis. All authors contributed to data interpretation. BA drafted the manuscript and MG and HB provided critical revision. All authors approved the final manuscript for submission.

## Figures and Tables

**Table 1:** Group characteristics before and after self-referential worry induction.

	Baseline group means (SDs)			One-way ANOVA
	Acceptance	Attention	PMR	
**Characteristics**				
Age	21.1 *(3.6)*	20.6 *(2.3)*	20.7 *(3.1)*	*F*_(2,70)_ = 0.38, *p* = 0.68
Gender	16F/7M	20F/6M	18F/6M	*χ*^*2*^ = 0.36*, p* = 0.83
Mindfulness Exposure	1.9 *(1.1)*	1.9 *(1.4)*	1.9 *(1.1)*	*F*_(2,70)_ = 0.07, *p* = .94^2^
Worry: likelihood	70.4 (20.4)	68.0 (20.9)	61.0 (22.6)	*F*_(2,70)_ = 1.27, *p* = 0.29
Worry: catastrophic	64.0 (20.3)	61.4 (17.2)	64.7 (22.8)	*F*_(2,70)_ = 0.18, *p* = 0.83
Worry: ability to cope	36.8 (25.2)	45.5 (22.0)	43.7 (23.2)	*F*_(2,70)_ = 0.91, *p* = 0.41
**State questionnaires**				
Anxiety (VAS)	23.2 (23.2)	25.7 (24.0)	24.1 (22.0)	*F*_(2,70)_ = 0.07, *p* = 0.93
Nervousness (VAS)	24.1 (21.0)	26.0 (22.0)	26.4 (20.6)	*F*_(2,70)_ = 0.08, *p* = 0.93
State Worry (VAS)	33.2 (27.1)	30.5 (19.8)	27.9 (19.3)	*F*_(2,70)_ = 0.34, *p* = 0.72
**Trait questionnaires**				
Anxiety (STAI)	44.2 *(13.2)*	43.2 *(11.7)*	47.8 *(12.1)*	*F*_(2,70)_ = 0.93, *p* = 0.40
Mindfulness (PMS)	60.9 *(7.8)*	61.7 *(7.9)*	61.7 *(6.8)*	*F*_(2,70)_ = 0.08, *p* = 0.93
Worry (PSWQ)	53.5 *(15.4)*	52.9 *(15.9)*	60.3 *(12.2)*	*F*_(2,70)_ = 1.93, *p* = 0.15
Attention Control (ACS)	44.9 *(8.0)*	46.0 *(7.4)*	46.0 *(5.7)*	*F*_(2,70)_ = 0.17, *p* = 0.84

**Table 2:** Mean *(SD)* frequency of thought intrusions before and after self-referential worry induction.

Thought intrusions	Pre-worry			Post-worry			Group x Time ANCOVA
	Acceptance	Attention	PMR	Acceptance	Attention	PMR	
Negative	0.70 *(1.02)*	0.88 *(0.86)*	0.67 *(0.64)*	1.04 *(1.22)*	1.81 *(1.47)*	2.54 *(1.79)*	*F*_(2,64)_ = 9.23, *p* < 0.001, *η*_*p*_^*2*^ = 0.22
Neutral	1.09 *(1.20)*	1.08 *(0.89)*	0.92 *(1.21)*	1.09 *(1.08)*	1.08 *(1.06)*	0.75 *(0.94)*	*F*_(2,64)_ = 0.38, *p* = 0.35
Positive	1.22 *(1.28)*	0.69 *(0.73)*	1.04 *(1.00)*	0.83 *(1.19)*	0.50 *(0.91)*	0.67 *(0.87)*	*F*_(2,64)_ = 0.11, *p* = 0.90

## Footnotes

1 There were no outliers on PSWQ across the sample, nor within each group. However removing high worriers (N = 2) from the PMR group and low worriers from acceptance (N = 4) and attention (N = 4) groups so that groups were better matched on PSWQ (M_acc_ = 55.0, M_att_ = 57.0, M_PMR_ = 58.6) did not alter the findings reported in the main text (Group × Time interaction on negative thought intrusions, *F*_(2,59)_ = 8.75, *p* < 0.001. Supplementary ANCOVAs controlling for small group differences in baseline PSWQ supported our primary findings and provided strong evidence of a group x time effect on negative intrusions that is consistent with the pattern shown in Fig. 1 [*F*_(2,64)_ = 7.34, *p* = 0.001] but not neutral or positive intrusions [*F*s < 0.42, *p*s > 0.66 – note limitations of ANCOVA in this regard, Miller & Chapman, 2001]. Finally, we conducted a sub-group analysis in high and low worriers (identified according to a threshold of PSWQ scores below/above 56, Hirsch et al., 2009, Hirsch et al., 2011). Acceptance and attention training reduced negative thought intrusions (vs. PMR) in both low/high trait worriers (*F*_(2,20)_ = 3.59, *p* = 0.047 and *F*_(2,37)_ = 4.68, *p* = 0.015, respectively). There was no evidence that trait worry moderated the effect of active worry on negative thought intrusions (Worry group x Time: *F*_(1,62)_ = 0.54, *p* = 0.46), nor the effect of the interventions on negative thought intrusions (Group x Worry group x Time, *F*_(2,62)_ = 0.45, *p* = 0.64).

2 Frequency of mindfulness practice was as follows: (1) I have never heard of mindfulness, N = 39. (2) I have heard of mindfulness, but I don't know what it is, N = 15; (3) I know what mindfulness is but have never tried it, N = 7; (4) I have tried meditation but not mindfulness, N = 9; (5) I have tried mindfulness meditation, N = 3; (6) I regularly practise mindfulness meditation, N = 4.
